# H_2_S inhibits high glucose-induced osteoblast injury by inhibiting ferroptosis in diabetic osteoporosis *in vitro*

**DOI:** 10.1590/1414-431X2025e14679

**Published:** 2025-11-14

**Authors:** Qingping Shi, Feihong Chen, Wen Wu

**Affiliations:** 1 Department of Endocrinology, Guangdong Geriatrics Institute, Guangdong Provincial People's Hospital (Guangdong Academy of Medical Sciences), Southern Medical University, Guangzhou, China; 2 Department of Osteoporosis, People's Hospital of Shenzhen Baoan District, The Second Affiliated Hospital of Shenzhen University, Shenzhen, China; 3 Department of Respiratory Medicine, People's Hospital of Shenzhen Baoan District, The Second Affiliated Hospital of Shenzhen University, Shenzhen, China

**Keywords:** High glucose, Osteoporosis, Hydrogen sulfide, Ferroptosis, Osteoblast

## Abstract

Diabetic osteoporosis (DOP) is a complication of prolonged hyperglycemia. Hydrogen sulfide (H_2_S) has been identified as a protective factor in bone development. However, the mechanism by which H_2_S antagonizes the effects of high glucose (HG) on osteoblasts remains unclear. The effects of HG and H_2_S on osteoblasts were assessed through transcriptomic and metabolomic sequencing to identify key changes in gene expression and metabolism. Reactive oxygen species (ROS) levels, mitochondrial membrane potential (MMP), alkaline phosphatase (ALP) activity, mineralization, iron ion levels, malondialdehyde (MDA) levels, cell proliferation, and protein expression were evaluated. Transcriptomic analysis revealed significant upregulation of the ferroptosis pathway in HG-treated osteoblasts. Fer-1 and H_2_S antagonized the HG-induced decrease in osteoblast cell proliferation, increase in ROS production, decrease in MMP, decrease in ALP, decrease in mineralized nodules, and increase in iron ions and MDA. Transcriptome analysis showed Fer-1 was involved in upregulating the synthesis, secretion, and action of parathyroid hormone and estrogen synthesis, while downregulating the mitogen-activated protein kinases (MAPK) pathway. Metabolomic analysis showed H_2_S restored glutathione metabolism, reducing pyroglutamic acid and L-5-oxoproline levels. Transcriptome sequencing identified downregulated genes (*hmox1*, *ncoa4*) and an upregulated gene (*slc40a1*) related to ferroptosis in the H_2_S + HG group compared with the HG group. Western blot analysis indicated H_2_S increased GPX4 and SLC7A11 levels while reducing ACSL4 expression compared with the HG group. Ferroptosis may be involved in the pathogenesis of DOP and H_2_S can effectively alleviate osteoblast injury by inhibiting ferroptosis in DOP.

## Introduction

Diabetic osteoporosis (DOP) is a skeletal disorder caused by prolonged hyperglycemia, leading to endocrine and metabolic disturbances. This condition results in bone matrix deterioration and progressive reduction in bone density, ultimately leading to an increased susceptibility to fractures (1). DOP is regarded as a chronic consequence of diabetes mellitus (DM) that impacts the musculoskeletal framework. At the core of abnormal bone metabolism in DOP lies the dysfunction of osteoblasts. Osteoblasts are crucial for bone formation as they are responsible for synthesizing, secreting, and mineralizing the bone matrix (2). The death of osteoblasts significantly impacts the bone formation process. The crucial function of osteoblasts in DOP has been emphasized in recent research. High glucose (HG) levels have been shown to upregulate the expression of divalent metal transporter 1, causing an excess of iron and oxidative stress damage in osteoblasts, ultimately resulting in impaired osteoblast function and decreased bone formation (3). However, the specific mechanisms underlying osteoblast damage in the diabetic microenvironment are still not fully understood.

Hydrogen sulfide (H_2_S), a transparent gas known for its distinctive rotten egg smell, acts as a gas transmitter in various signaling pathways (4,5). H_2_S plays a vital part in multiple physiological processes, including triggering vascular calcification, promoting cell growth, regulating blood pressure, responding to inflammation, facilitating autophagy, suppressing carcinoma, and influencing age-related alterations (6-8). The development of different human diseases, including diabetic nephropathy, diabetic cardiomyopathy, osteoporosis, and osteoarthritis, has been associated with abnormal H_2_S metabolism (9-11). H_2_S participates in diverse cellular signaling pathways, exerting beneficial effects such as anti-inflammatory, cell-protective, antioxidant, and skeletal growth processes (12). In recent years, the protective effects of H_2_S on bone have attracted significant attention from researchers (13). Given that oxidative harm plays a key role in the morphological and functional changes observed during the progression of DOP, the antioxidant properties of H_2_S suggest its potential to protect against DOP development (14).

Nevertheless, it remains uncertain if H_2_S has a defensive function in the damage of osteoblasts in DOP. This study aimed to elucidate the mechanisms by which H_2_S exerts its effects on osteoblast damage in DOP.

## Materials and Methods

### Substances and chemicals

Fetal bovine serum (FBS), Trizol, and Gibco minimum essential medium α (α-MEM) were procured from Thermo Fisher Scientific, Inc. (USA). GYY4137 (H_2_S donor), ferroptosis inhibitor (Fer-1), and rhodamine 123 (Rh123) were acquired from Sigma (USA). ALP kit and 2',7'-dichlorofluorescein diacetate (DCFH-DA) were obtained from Nanjing Jiancheng Bioengineering Institute (China). Alizarin red was purchased from Yuan Ye Biological Company (China). The iron ion colorimetric assay kit and RIPA lysis buffer were procured from Beijing Apply Gene Technology Company (China). The malondialdehyde (MDA) detection kit was obtained from Abbkine Scientific Company (China). GPX 4(67763-1-Ig), ACSL4 (22401-1-AP), and β-actin (66009-1-Ig) were sourced from Proteintech Group, Inc, (USA). SLC7A11 was purchased from ABclonal Technology Co., Ltd. (China). Polyclonal goat anti-rabbit secondary antibody was obtained from Cell Signaling Technology, Inc. (USA).

### Cultivation and treatment of osteoblasts

The experiment utilized the MC3T3-E1 Subclone 14 cell line derived from mouse osteoblasts, which were acquired from the National Collection of Authenticated Cell Cultures (China). This study comprised a total of six experimental groups: control, HG, H_2_S+HG, H_2_S, Fer-1+HG, and Fer-1 groups.

### Assessment of cellular viability

The viability of osteoblasts in mice was evaluated according to the guidelines provided by the cell counting kit-8 assay kit (CCK-8) (NU679, Dojindo Laboratories, Japan). The CCK-8 quantifies cell viability based on the reduction of WST-8 by cellular dehydrogenases to form a water-soluble orange formazan product, with absorbance proportional to the viable cell number (15). Absorbance was measured at a wavelength (λ) of 450 nm.

### Intracellular ROS detection

Mouse osteoblasts were cultured with 10 μM DCFH-DA for 30 min, followed by PBS washing. Fluorescent light was detected by a fluorescence microscope (Nikon, Japan). Intracellular ROS was indicated by the levels of mean fluorescence intensity (MFI). The measurement was performed utilizing the ImageJ application (1.8.0 version, NIH, USA). Each experiment was repeated in triplicate.

### Intracellular MMP examination

Mouse osteoblasts were co-incubated with Rh123 (2 μM) for 45 min. Subsequently, osteoblasts were rinsed with PBS. Fluorescent mitochondria were observed using a fluorescence microscope. MMP levels were displayed using MFI. Analysis was performed using ImageJ software (NIH). The experiment was repeated in triplicate.

### Alkaline phosphatase assay

Following the incubation of mouse osteoblasts in osteogenic induction medium for 7 days, the medium was removed. RIPA lysis buffer was used to completely lyse osteoblasts for 30 min on ice. Next, the lysates were spun at 4°C utilizing a low-temperature centrifuge (Scilogex, USA) for 10 min. The liquid above the sediment was gathered and the activity of alkaline phosphatase (ALP) was determined following the guidelines provided in the ALP assay kit. Absorbance was measured at a λ of 520 nm. With a BCA protein quantification kit (Sigma), we tested the protein levels in each sample. By utilizing the absorbance values and protein concentrations, we calculated the cellular ALP activity of osteoblasts based on the instructions provided in the ALP assay kit.

### Alizarin red staining of calcified nodules

Following a 21-day culture of osteoblasts in osteogenic induction medium, the process of alizarin red staining was carried out. Orange-red calcified nodules of osteoblasts were observed when examined under a microscope. To chelate with calcium nodules, 10% hexadecyl pyridinium chloride was used to dissolve alizarin red. A 620-nm wavelength was used to detect the absorbance.

### Metabolomic analyses

Ultra-performance liquid chromatography (SCIEX, USA) was employed in this experiment. The column for chromatographic analysis was kept at a temperature of 35°C while maintaining a flow rate of 0.4 mL per minute. Mobile phase A was composed of water containing 0.1% formic acid, while mobile phase B was comprised of acetonitrile with 0.1% formic acid.

For mass spectrometry analysis, a high-resolution mass spectrometer (SCIEX) was used. Each sample was analyzed twice, once for positive ions and once for negative ions. The ion source's shielding pressure was set at 30 pounds per square inch, and both auxiliary gas and sheath gas were maintained at 60 pounds per square inch. The voltage for detecting positive ions was adjusted to 5000 v, while the voltage for detecting negative ions was set to 4500 v. Throughout the process of data acquisition, the accuracy of the instrument was calibrated after every 20 samples. Furthermore, a scan of a quality control (QC) sample was conducted following every set of 10 samples.

### Transcriptomic analyses

Each group consisted of three samples. Following the manufacturer's instructions, TRizol was employed to extract total RNA. Subsequently, for the construction of cDNA libraries, 1 µg of extracted RNA was utilized. Invitrogen SuperScript™ II Reverse Transcriptase (Catalog number 1896649, USA) was used to synthesize cDNA. The constructed transcriptomic libraries were subjected to Illumina Novaseq™ 6000 sequencing conducted by LC-Bio Technology Co., Ltd. (China). Genes exhibiting a fold change (FC) above 2 or below 0.5, along with a P value below 0.05, were identified as differentially expressed genes (DEGs). The DEGs underwent enrichment analysis by Gene Ontology (GO) and Kyoto Encyclopedia of Genes and Genomes (KEGG).

### Iron ions and malondialdehyde (MDA)

The iron ion assay was conducted following the guidelines provided by the manufacturer, utilizing the iron ion detection kit. Measurement of absorbance was carried out at λ of 620 nm. The concentration of iron ions was determined by utilizing the standard curve.

MDA detection was performed using the MDA assay kit following the manufacturer's instructions. The measurement of absorbance was conducted at wavelengths of 532 and 600 nm. By calculating the difference in absorbance measurements between these two wavelengths, the MDA levels were determined.

### Western blot (WB) analysis

Cell lysates were extracted by RIPA buffer, and the protein content was quantified via a BCA assay. Protein samples were equally loaded onto SDS-PAGE gels for separation and then transferred onto PVDF membranes. The membranes were blocked with 5% non-fat milk in TBST for 1 h at room temperature before being incubated overnight at 4°C with primary antibodies targeting specific proteins. The following day, membranes were rinsed with TBST and then treated with HRP-linked secondary antibodies for 1 h at room temperature. Protein bands were detected using an ECL substrate and visualized using a chemiluminescence imaging system (Bio-Rad, USA).

### EdU cell proliferation assay

Osteoblast proliferation was assessed using the Click-iT EdU assay kit (Servicebio, China). Osteoblasts were incubated with 20 μM EdU for 2 h, followed by fixation and staining according to the manufacturer's protocol. MFI of EdU signal was quantified using ImageJ software (NIH) by measuring integrated density in ≥100 cells, normalized to background.

### Statistical analysis

To analyze the experiment data and visualize it graphically, we used the GraphPad Prism 8.0.2 software (GraphPad Software Inc., USA). For comparative analyses, at least three independent replicates were performed for each experiment. Comparisons among multiple groups were conducted by one-way ANOVA, followed by Tukey's test. The data are reported as means±SD or means±SE. Statistical significance is defined as P<0.05.

## Results

### HG-induced osteoblast damage

**Figure 1:**
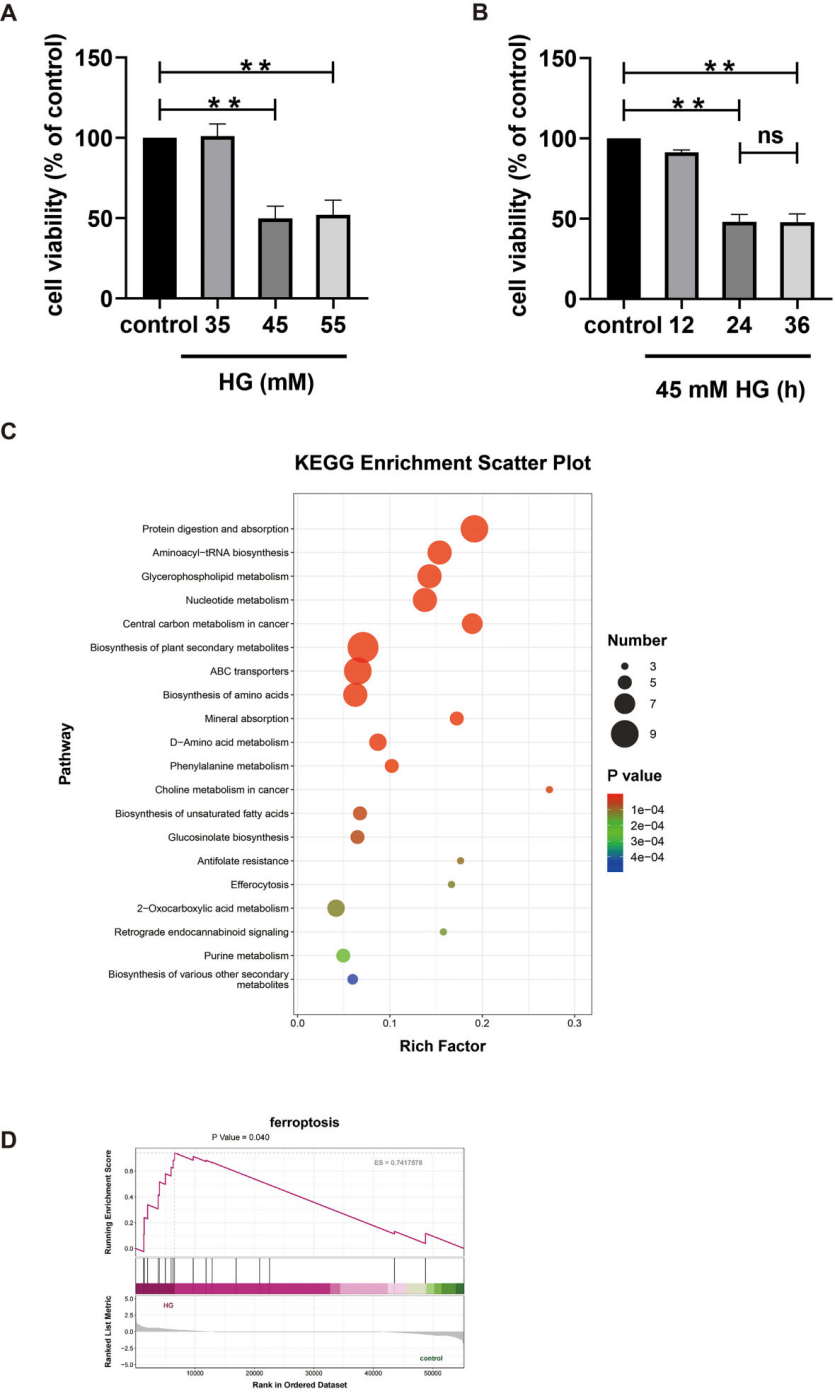
Effects of high glucose (HG) on MC3T3-E1 cells. **A**, Viability of MC3T3-E1 cells treated with different concentrations of HG by CCK 8. **B**, Viability of MC3T3-E1 cells treated with HG for different durations by CCK 8. **C**, KEGG enrichment scatter plot of metabolomic analysis of MC3T3-E1 cells treated with HG. **D**, Transcriptomic analysis of the ferroptosis pathway in HG-induced cytotoxicity in MC3T3-E1 cells. Data are reported as means±SD, n=3. **P<0.01; ns: P>0.05 (ANOVA).

To assess the cytotoxic effects of HG on MC3T3-E1 cells, different concentrations and exposure durations were evaluated. As illustrated in Figure 1A, MC3T3-E1 cells were treated with increasing concentrations of HG for a uniform duration of 24 h. Results indicated that 35 mmol/L HG did not significantly affect cell viability (P>0.05). However, at concentrations of 45 and 55 mmol/L, HG exhibited marked cytotoxicity (P<0.01), reducing cell viability by approximately 50% compared to the control group, with no significant difference between the two concentrations (P>0.05). Consequently, 45 mmol/L HG was selected for further time-response studies. Exposure to 45 mmol/L HG for 24 and 36 h revealed substantial cytotoxic effects, with the most pronounced decrease in cell viability observed at 24 h (P<0.01, Figure 1B). No statistically significant difference (P>0.05) in cytotoxicity markers was observed between 24 and 36 h. Based on these findings, subsequent experiments were conducted with MC3T3-E1 cells exposed to 45 mmol/L HG for 24 h.

Metabolomic analysis of the effects of HG on MC3T3-E1 cells revealed that the differential metabolites are primarily involved in the following signaling pathways: glycerophospholipid metabolism, nucleotide metabolism, ABC transporters, biosynthesis of amino acids, mineral absorption, phenylalanine metabolism, biosynthesis of unsaturated fatty acids, 2-oxocarboxylic acid metabolism, and retrograde endocannabinoid signaling (Figure 1C).

Transcriptomic analysis of the effects of HG on MC3T3-E1 cells revealed that the ferroptosis pathway is significantly upregulated in the HG group, as indicated by Gene Set Enrichment Analysis (GSEA) analysis (P<0.05) (Figure 1D). This suggests that ferroptosis may play a role in HG-induced cytotoxicity in MC3T3-E1 cells.

### Ferroptosis mediated HG-induced osteoblast damage

**Figure 2:**
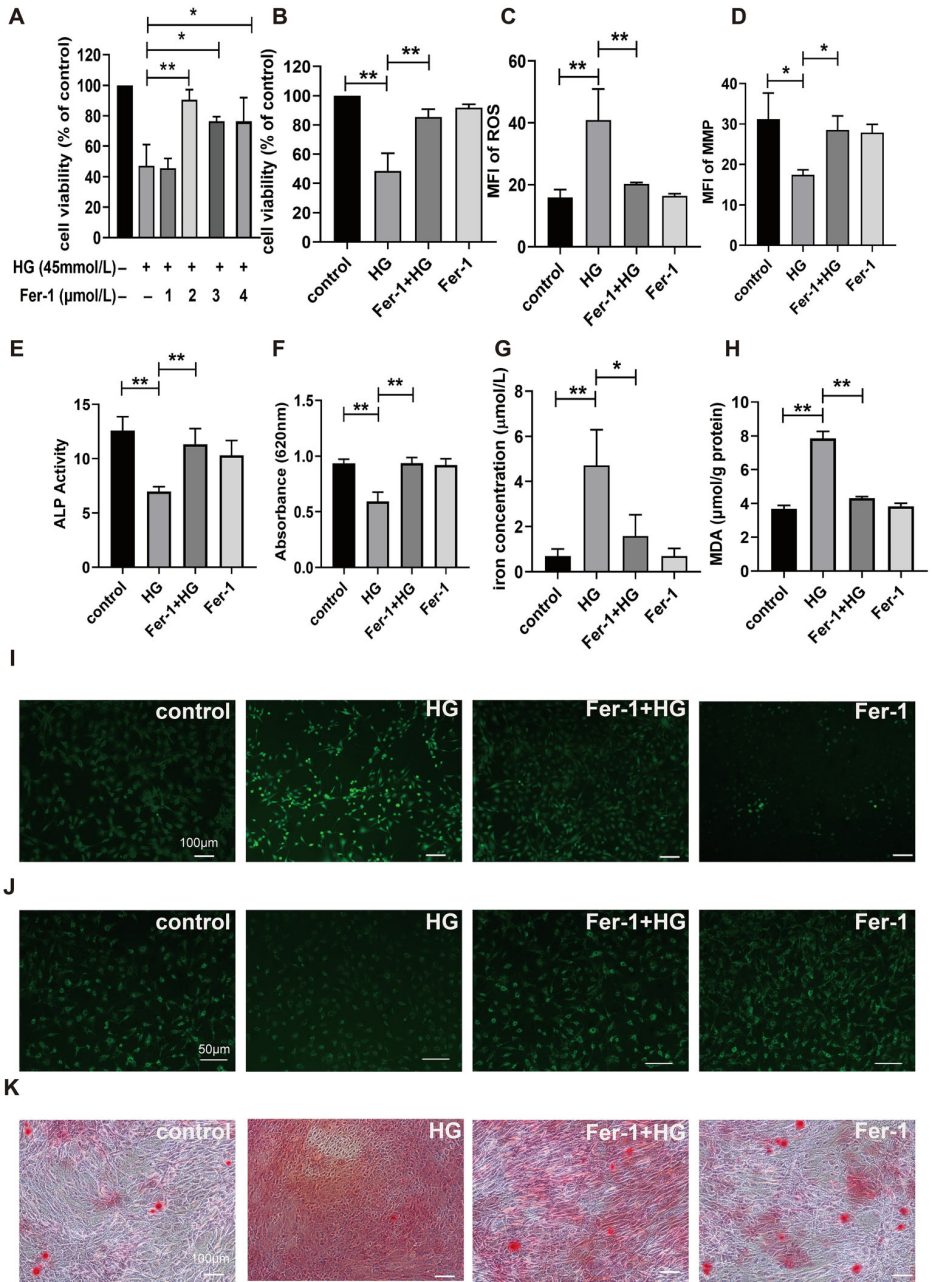
Fer-1 antagonizes high glucose (HG)-induced damage in osteoblasts. **A**, Cell viability in osteoblasts treated with different concentrations Fer-1 by CCK 8. **B**, Cell viability in osteoblasts treated with HG or Fer-1 by CCK 8. **C**, Mean fluorescence intensity (MFI) of reactive oxygen species (ROS) and **D**, Rh123 in osteoblasts. **E**, Quantitative analysis of alkaline phosphatase (ALP), **F**, alizarin red staining, **G**, iron ion, and **H**, malondialdehyde (MDA) in osteoblasts. **I**, Representative fluorescence images of ROS (scale bar=100 μm) and **J**, mitochondrial membrane potential (MMP) (scale bar=50 μm). **K**, Images of alizarin red staining in osteoblasts (scale bar=100 μm). Data are reported as means±SD; n=3. *P<0.05, **P<0.01 (ANOVA).

Cell viability was measured at different concentrations of Fer-1 and detected using the CCK-8 assay to investigate the effect of Fer-1 in osteoblasts. As shown in Figure 2A, we co-cultured osteoblasts with different concentrations of Fer-1 (0, 1, 2, 3, and 4 μmol/L) in cell culture medium with 45 mmol/L HG. The results showed that 2, 3, and 4 μmol/L Fer-1 had a significant effect on cell viability when co-cultured with HG (P<0.05). Fer-1 (2 μmol/L) had the greatest effect on cell viability (P<0.01). Based on the above results, when 2 μmol/L Fer-1 was co-cultured with 45 mmol/L HG for 24 h in osteoblasts in the subsequent experiments, the cell survival rate increased in comparison to the HG group (P<0.01; Figure 2B). The above experiments indicated that Fer-1 significantly alleviated HG cytotoxicity in osteoblasts.

Figure 2C and I demonstrate that the addition of HG resulted in a 2.6-fold rise in the MFI of intracellular DCFH in osteoblasts, significantly surpassing the control group (P<0.01). After co-culturing with Fer-1 and HG, the MFI value of intracellular DCFH was reduced two times compared with the HG group (P<0.01). However, adding Fer-1 alone had no notable impact on intracellular ROS in osteoblasts (P>0.05). These results indicated that Fer-1 counteracted HG-induced ROS generation in osteoblasts. Since Fer-1 can reduce ROS levels, we investigated whether it could improve mitochondrial damage by examining intracellular MMP levels. As shown in Figure 2D and J, after HG addition, the intracellular MFI of Rh123 decreased 1.8 times (P<0.05). However, after co-culturing with Fer-1 and HG, the intracellular MFI of Rh123 increased by 1.5 times (P<0.05) compared to the HG group. When Fer-1 was added alone, there was no significant effect on intracellular MMP in osteoblasts (P>0.05). The findings indicate that Fer-1 inhibited the HG-induced reduction in MMP in osteoblasts. Afterward, we investigated the impact of Fer-1 on the process of osteogenic differentiation through the examination of ALP activity and alizarin red staining. As shown in Figure 2E, after culture with HG, the ALP activity of osteoblasts was significantly decreased compared with the control group, while co-culture with Fer-1 and HG resulted in a significant increase in ALP activity compared to the HG group (P<0.01). This suggests that Fer-1 antagonized the HG-induced decrease in the osteogenic differentiation of osteoblasts.

**Figure 3:**
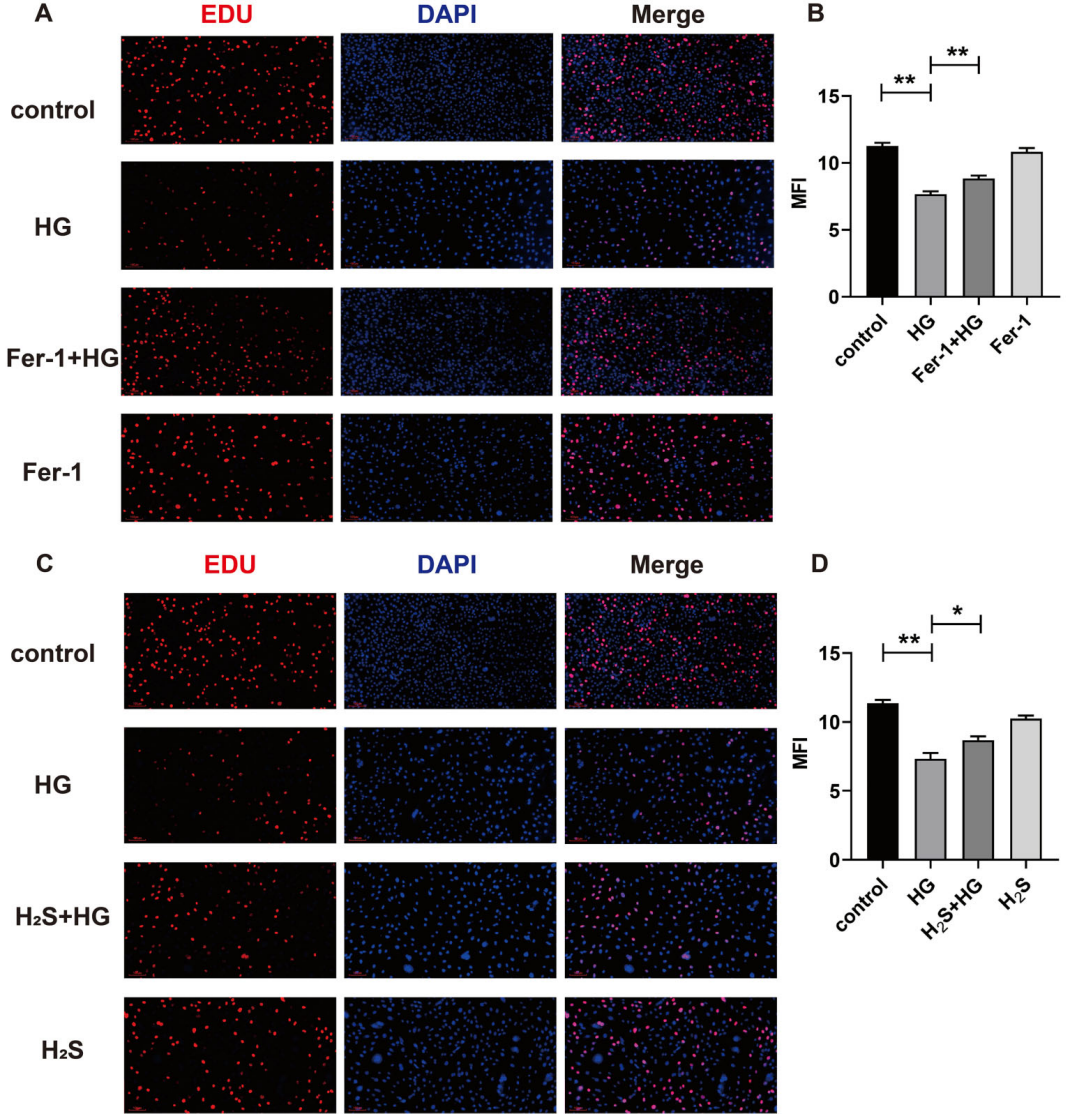
Comparative effects of H_2_S and Fer-1 on osteoblast proliferation under high glucose (HG) conditions. **A** and **C**, Representative fluorescence images of EdU staining (red) and nuclear counterstaining (Hoechst 33342, blue) in different treatment groups. Scale bar: 100 μm. **B** and **D**, Quantitative analysis of mean fluorescence intensity (MFI). Data are reported as means±SD; n=3. *P<0.05, **P<0.01 (ANOVA).

Figure 2F and K show the results of quantitative analysis and microscopic images after Alizarin red staining. The number of calcium nodules decreased after the addition of HG but increased after the addition of Fer-1. Enzyme-linked immunosorbent assay results showed that the number of mineralized nodules in osteoblasts co-cultured with Fer-1 and HG was 1.6 times higher than that in cells cultured with HG alone. This suggests that Fer-1 can improve the decrease of ALP activity and mineralized nodules in osteoblasts induced by HG. As shown in Figure 2G, after adding Fer-1, the concentration of iron ions decreased by approximately 3 times that of the HG group (P<0.01). According to Figure 2H, the MDA content in the HG group was significantly higher than that in the control group. However, after adding Fer-1, the MDA content significantly decreased (P<0.01). This suggests that Fer-1 inhibits the accumulation of iron and MDA induced by HG. As shown in Figure 3A and B, HG exposure significantly reduced EdU incorporation, evidenced by decreased MFI (P<0.01). Fer-1 treatment partially restored proliferation (P<0.01), while Fer-1 alone maintained MFI at control levels (P>0.05). These results indicate that Fer-1 protected osteoblasts from HG-induced proliferation suppression.

### Transcriptome analysis of Fer-1 antagonizing HG-induced osteoblast injury

**Figure 4:**
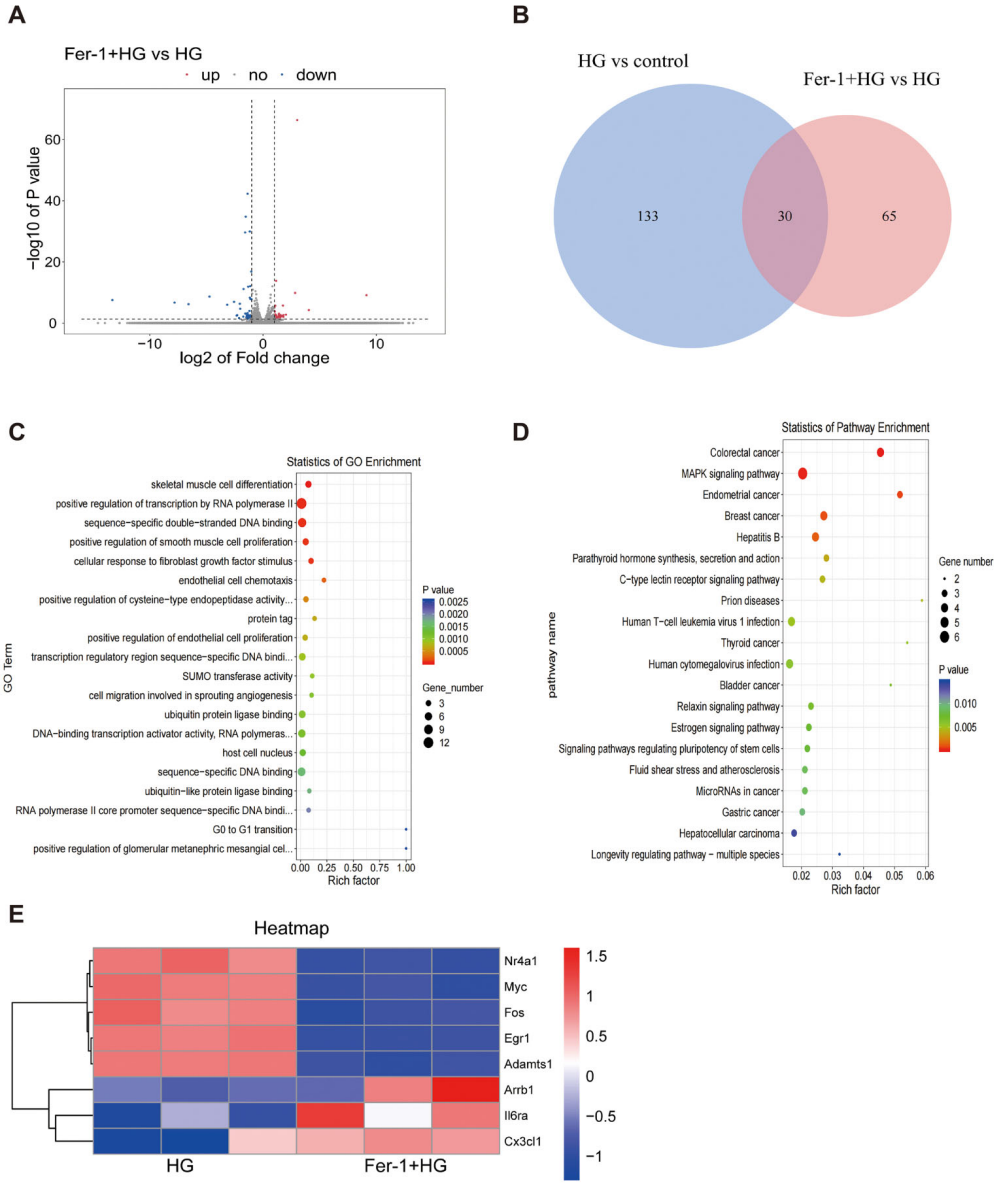
Analysis of differentially expressed genes (DEGs) after adding Fer-1. **A**, Volcano plot of DEGs in osteoblasts from the co-cultured Fer-1 and high glucose (HG) group. **B**, Venn diagram. **C**, Gene Ontology (GO) enrichment analysis. **D**, Kyoto Encyclopedia of Genes and Genomes (KEGG) pathway enrichment analysis. **E**, Cluster analysis of genes related to ferroptosis pathway (heatmap). n=3.

Compared to the HG group, the Fer-1 and HG co-culture group had 95 DEGs, with 42 showing upregulation and 53 showing downregulation (Figure 4A). Venn diagram analysis showed 30 common DEGs in the HG group (Figure 4B). GO bubble plot analysis revealed that Fer-1 enhanced the differentiation of skeletal muscle cells and the proliferation of smooth muscle cells (Figure 4C), and cluster analysis (heatmap) of DEGs related to these two biological processes is shown in Figure 4E. KEGG analysis showed that Fer-1 was involved in upregulating the synthesis, secretion, and action of parathyroid hormone and estrogen synthesis, while downregulating the mitogen-activated protein kinases (MAPK) pathway (Figure 4D).

### H_2_S antagonized the HG-induced damage in osteoblasts

**Figure 5:**
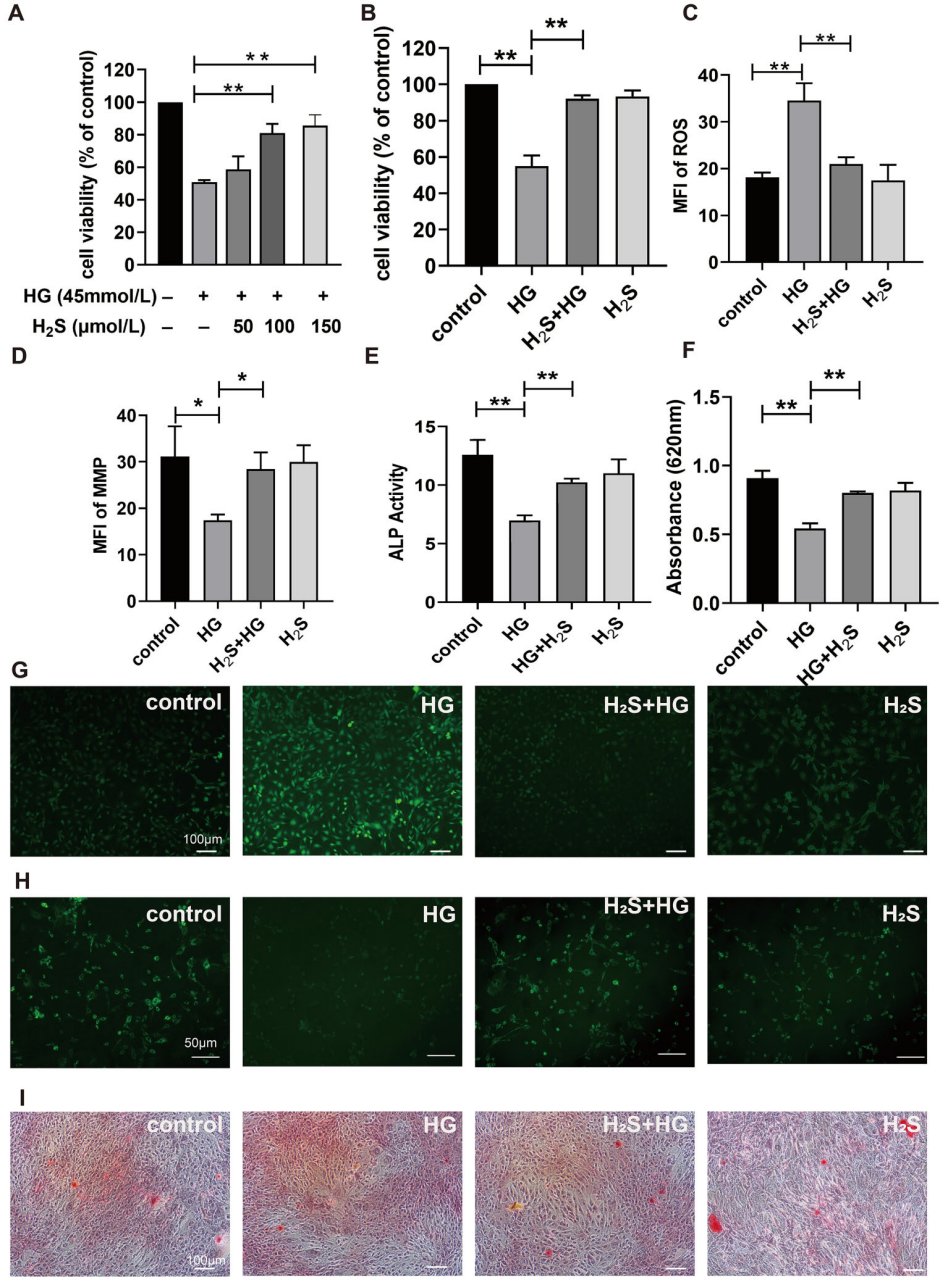
H_2_S antagonizes high glucose (HG)-induced damage in osteoblasts. **A**, Cell viability in osteoblasts treated with different concentrations analyzed by CCK 8. **B**, Cell viability in osteoblasts treated with HG or H_2_S by CCK 8. **C**, Mean fluorescence intensity (MFI) of reactive oxygen species (ROS) and **D**, Rh123 in osteoblasts. **E**, Quantitative analysis of alkaline phosphatase (ALP) and **F**, alizarin red staining in osteoblasts. **G**, Representative fluorescence images of ROS (scale bar=100 μm) and **H**, mitochondrial membrane potential (MMP) in osteoblasts (scale bar=50 μm). **I**, Alizarin red staining images in osteoblasts (scale bar=100 μm). Data are reported as means±SD; n=3. *P<0.05, **P<0.01 (ANOVA).

To assess the impact of H_2_S on osteoblasts, cell viability was evaluated at varying concentrations of H_2_S using the CCK-8 assay. As shown in Figure 5A, osteoblasts were cultured with different GYY4137 (an H_2_S donor) concentrations (0, 50, and 150 μmol/L) with HG. The findings revealed that GYY4137 at 100 and 150 μmol/L significantly influenced cell viability in the presence of HG (P<0.01). The restorative effect of 100 μmol/L GYY4137 on HG-induced osteoblast damage was statistically significant and showed no notable difference compared to 150 μmol/L. Therefore, we selected 100 μmol/L as the experimental concentration of GYY4137.

To investigate the exogenous H_2_S antagonism of HG-induced cytotoxicity in osteoblasts, we conducted CCK-8 experiments. As shown in Figure 5B, after osteoblasts were cultured with HG, cell viability decreased by approximately 50% (P<0.01) compared to the control group. However, when 100 µmol/L of GYY4137 was added, we observed that exogenous H_2_S significantly alleviated HG-induced cytotoxicity. As shown in Figure 5C and G, after osteoblasts were cultured with HG for 24 h, the MFI (reflecting intracellular ROS) of DCFH increased, and the difference between the HG and the control group was significant (P<0.01). However, when exogenous H_2_S and HG were co-cultured for 24 h, the increase in ROS generation in osteoblasts induced by HG was antagonized, and the MFI of DCFH decreased compared to that in the HG group (P<0.01).

Oxidation-reduction reactions can lead to mitochondrial damage in cells. We assessed the degree of mitochondrial damage by measuring the MFI (reflecting intracellular MMP) of Rh 123. As shown in Figure 5D and H, following HG addition to osteoblasts, the MFI value of Rh123 decreased compared to that of the control (P<0.05). However, the HG-induced decrease in MMP production in osteoblasts was antagonized after co-culturing the cells with exogenous H_2_S and HG (P<0.05). In contrast, the addition of exogenous H_2_S alone did not have a notable impact on the intracellular MMP in osteoblasts (P>0.05).

The activity of ALP, a crucial enzyme in osteoblasts, is directly linked to the formation of bone. As shown in Figure 5E, ALP activity in the HG group was markedly reduced compared to the control group (P<0.05). When exogenous H_2_S was added, the ALP value increased in contrast to the HG group (P<0.05), whereas the addition of exogenous H_2_S alone had no significant effect on ALP (P>0.05). Figure 5I shows microscopic images of Alizarin red S staining. The number of calcium nodules decreased after adding HG, whereas the number of calcium nodules increased after adding exogenous H_2_S. Additionally, a quantitative analysis of Alizarin red S staining was conducted, as depicted in Figure 5F, and the results aligned with the microscopic staining findings. After HG treatment, the number of mineralization nodules decreased, whereas co-culture with exogenous H_2_S and HG increased the number of mineralization nodules compared to the HG group (P<0.01). Treatment with exogenous H_2_S alone had no significant impact on osteoblasts mineralization (P>0.05). As shown in Figure 3C and D, exposure to HG significantly reduced EdU incorporation (P<0.01). H_2_S treatment partially reversed this suppression (P<0.05), while H_2_S alone maintained normal MFI levels (P>0.05). These data demonstrate that H_2_S mitigated HG-induced proliferation inhibition in osteoblasts.

### Metabolomics analysis of H_2_S counteracting HG-induced osteoblast injury

**Figure 6:**
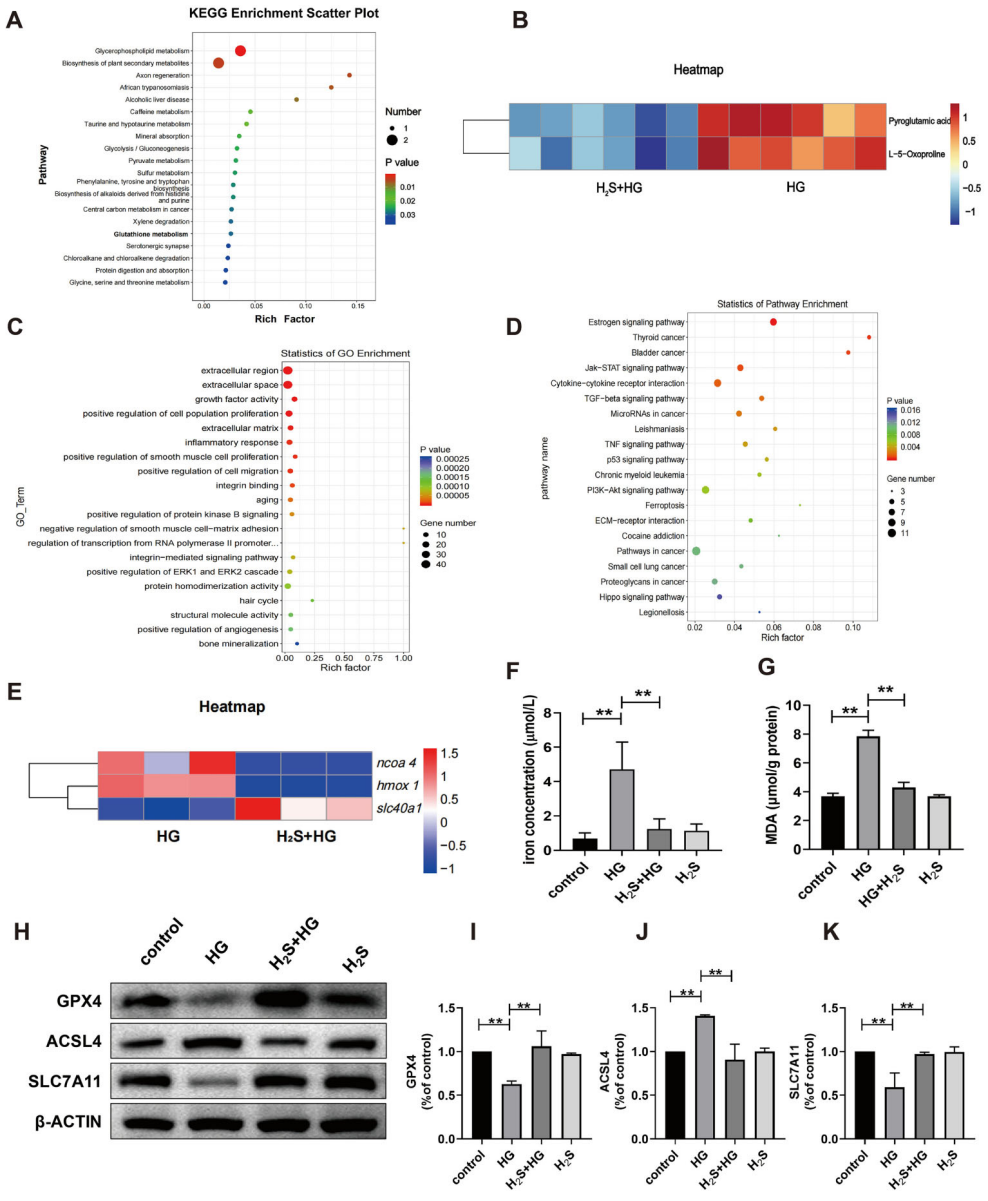
Metabolomic and transcriptomic analyses show that H_2_S inhibited high glucose (HG)-induced injury in osteoblastic cells. **A**, Analysis of differential metabolite KEGG pathway caused by H_2_S. **B**, Metabolomic heatmap analysis of H_2_S+HG and HG group. **C**, Gene Ontology (GO) enrichment analysis. **D**, KEGG pathway enrichment analysis. **E**, Cluster analysis of genes related to ferroptosis pathway (heatmap). **F**, Quantitative analysis of iron and **G**, malondialdehyde (MDA) in osteoblasts caused by H_2_S. **H**, WB analysis of the expression levels of ferroptosis-related proteins (GPX4, ACSL4, and SLC7A11) in MC3T3-E1 cells. **I**, Quantitative analysis of GPX4 expression, **J**, ACSL4 expression, and **K**, SLC7A11 expression. Data are reported as means±SD; n=3. **P<0.01 (ANOVA).

KEGG analysis of differential metabolites revealed that H_2_S was involved in various metabolic pathways, including purine metabolism, glutathione metabolism, glycerophospholipid metabolism, and mineral absorption (Figure 6A).

Metabolomic analysis has revealed that H_2_S regulates glutathione metabolism, which is crucial for preventing ferroptosis. Specifically, in HG conditions, the levels of pyroglutamic acid and L-5-oxproline are elevated (Figure 6B).

The increase in pyroglutamic acid and L-5-oxproline in the HG group suggests impaired glutathione metabolism, leading to reduced antioxidant capacity and increased susceptibility to ferroptosis. Conversely, the decrease in these metabolites in the H_2_S+HG group indicates that H_2_S helps restore glutathione metabolism, enhancing antioxidant defenses and reducing ferroptosis.

### Transcriptome analysis of H_2_S attenuated HG-induced osteoblast injury

As shown in Figure 6C, the bubble plot of the GO enrichment analysis results shows that exogenous H_2_S has positive effects on cell proliferation, smooth muscle cell proliferation, cell migration, and bone mineralization, while inhibiting inflammation and aging. In KEGG enrichment analysis, we observed that exogenous H_2_S affected multiple biological signaling pathways (Figure 6D). Specifically, exogenous H_2_S upregulated the estrogen signaling pathway and downregulated the P53, PI3k-AKT, and ferroptosis signaling pathways. The role of the ferroptosis signaling pathway drew our attention. We found exogenous H_2_S downregulated the expression of heme oxygenase 1 (*hmox 1*) and nuclear receptor coactivator 4 (*ncoa 4*), while upregulating the expression of solute carrier family 40 member 1/iron-regulated transporter member 1 (*slc40a1*) (Figure 6E) compared with the HG group.

### H_2_S counteracted the increase in iron ions and MDA caused by HG in osteoblasts

In the ferroptosis pathway, ferrous ions accumulate in large quantities inside the cells and undergo lipid peroxidation via the Fenton reaction. The increase in lipid peroxidation products is a unique biological feature of ferroptosis, which can be reflected by detecting lipid peroxidation products. At present, MDA is acknowledged as the outcome of lipid peroxidation metabolism, and its quantity directly indicates the amount of lipid peroxide within the cells. According to the data presented in Figure 6F and G, the HG group had significantly higher iron ion and MDA contents than the control group (P<0.01). However, the addition of exogenous H_2_S resulted in a significant decrease in iron ion concentration and MDA content (P<0.01). This indicated that HG causes iron metabolism disorders and lipid peroxidation, whereas exogenous H_2_S antagonizes iron accumulation and increases MDA caused by HG.

### H_2_S modulated ferroptosis-related expression

In the HG group, glutathione peroxidase 4 (GPX4) expression was notably decreased. However, in the H_2_S+HG group, GPX4 expression increased compared to the HG group (Figure 6H and I). Conversely, Acyl-CoA synthetase long-chain family member 4 (ACSL4) expression was elevated in the HG group, while it was reduced in the H_2_S+HG group relative to the HG group (Figure 6H and J). Additionally, Solute Carrier Family 7 Member 11 (SLC7A11) expression declined in the HG group but was significantly elevated in the H_2_S+HG group (Figure 6H and K).

### Analysis of transcriptome and metabolome in osteoblasts

Furthermore, the KEGG pathway enrichment analysis of the transcriptome and metabolome of the HG and H_2_S+HG groups is shown in Supplementary Figure S1A. The genes and their corresponding metabolites were enriched in endocrine and other factor-regulated calcium reabsorption, CAMP signaling pathway, AMPK signaling pathway, fructose and mannose metabolism, and purine metabolism. Network analyses showed that soluble carrier family 40 member 1 (*slc40a1*) and *hmox1* were related to L-tryptophan (Supplementary Figure S1B).

## Discussion

Globally, diabetes affects approximately one in every 11 adults, and osteoporosis is often comorbid with diabetes, which is influenced by various factors. Two factors that impact bone formation are the accumulation of advanced glycation end products in collagen and oxidative stress resulting from elevated blood glucose levels. Furthermore, the occurrence of osteoporosis has been associated with reduced levels of insulin and insulin-like growth factor-1. Anti-diabetic drugs, such as thiazolidinediones, have been linked to adverse impacts on bone metabolism and an increased risk of fractures. Nevertheless, the comprehensive pathophysiological mechanisms of DOP remain incompletely understood. A deeper understanding of the pathogenesis of DOP could improve its prediction and facilitate timely and suitable prevention and management of osteoporotic fragility fractures.

Our findings demonstrate that bone cells exposed to 45 mmol/L HG for 24 h have significantly reduced osteoblast viability, consistent with previous studies highlighting the cytotoxic effects of hyperglycemia on bone cells (16). Metabolomic analysis revealed significant disruptions in glycerophospholipid metabolism, nucleotide metabolism, and amino acid biosynthesis pathways, implicating oxidative stress and ferroptosis in HG-induced damage. Specifically, the upregulation of ferroptosis-related pathways in HG-treated cells underscores the pivotal role of ferroptosis in osteoblast cytotoxicity under hyperglycemic conditions.

Ferroptosis is associated with various diseases, and research on its relationship with osteoporosis has gained widespread attention. According to Lu et al. (17), glucocorticoids can activate ferroptosis in osteoblasts through the downregulation of GPX4, elevating MDA and ROS levels, and inhibiting cysteine production in a DOP mouse model. One study highlights the role of the METTL3/ASK1-p38 signaling pathway in activating osteoblast ferroptosis under high glucose and high fat conditions, which are common in diabetic osteoporosis (18). Clinical studies suggest that iron metabolism disorders are common in diabetic patients (19). Iron is a powerful oxidizing agent that can stimulate the generation of different ROS. Indicators related to iron metabolism (transferrin, ferritin, and transferrin receptor) have the potential to directly or indirectly impact the onset and progression of DM (19). In a study on DOP using rats, Wang et al. (20) found that treatment of osteoblasts with HG decreased GPX4 expression, increased ROS levels, and led to the accumulation of lipid peroxides. However, melatonin significantly reduced HG-induced ferroptosis by activating the Nrf2/HO-1 signaling pathway both *in vitro* and *in vivo* and enhancing the osteogenic ability of MC3T3-E1 cells. The findings suggest that HG stimulates ferroptosis in osteoblasts. The development of DOP could be strongly associated with ferroptosis. As H_2_S has antioxidant effects, its role in ferroptosis has been investigated. Previous studies have shown that H_2_S controls the balance of iron within cells by impacting the storage and transportation of iron (21). Also, the role of H_2_S in activating Nrf2 and PPAR-γ to reduce oxidative stress and inflammation and inhibit ferroptosis has been identified (22,23).

Additionally, reverse validation using the ferroptosis inhibitor Fer-1 showed that it counteracted the HG-induced decrease in osteoblast activity, increase in ROS generation, decrease in MMP production, decrease in ALP, decrease in mineralized nodules, increase in iron accumulation, increase in MDA levels, and proliferation inhibition. Excessive iron has been linked to bone loss in mice, which is closely associated with osteoporosis. Earlier research has indicated that iron inhibits the osteogenic differentiation of bone marrow stromal cells, and excessive iron in mice is linked to higher amounts of iron protein and lower amounts of RUNX family transcription factor 2 in osteoprogenitor cells (24). The study revealed that Fer-1 enhances the differentiation of skeletal muscle cells and promotes the proliferation of smooth muscle cells according to transcriptional GO analysis. Additionally, KEGG analysis indicated that Fer-1 plays a role in upregulating the synthesis and secretion of parathyroid hormone and estrogen and downregulating the MAPK pathway. Research has indicated that the administration of Fer-1 hinders lipid peroxidation caused by HG in osteoblasts, suppresses ferroptosis, and enhances the condition of osteoporosis (25). Both parathyroid hormone and estrogen promote osteoblasts differentiation (26). ROS production is linked to the MAPK pathway, and Poursaitidis et al. (27) showed that inhibiting the MAPK signaling pathway can decrease the effects of ferroptosis in lung cancer cells. Similarly, downregulation of the MAPK pathway (especially p38 and JNK) can inhibit ferroptosis in AML cells (28).

In this study, we found that H_2_S alleviated HG-induced cytotoxicity, increased ROS generation, decreased MMP production, reduced ALP levels, and decreased mineralized nodules and proliferation inhibition *in vitro*. These findings suggest a protective effect of H_2_S against HG-induced osteoblasts injury. Previous research has demonstrated that H_2_S mitigates the decrease in cell proliferation, the increase in apoptosis, and the reduced mineralization in osteoblasts caused by HG (14). Furthermore, the protective effects of H_2_S against oxidative stress-induced cytotoxicity are also supported by its role in modulating signaling pathways. For instance, H_2_S protects against oxidative damage by activating the Nrf2 pathway, enhancing antioxidant expression to reduce ROS in osteoblasts under HG conditions (29). The findings from these experiments provided evidence for the beneficial effect of H_2_S in preventing osteoblast injury caused by HG. However, the protective mechanisms of H_2_S in this process remain unclear. Therefore, we employed metabolomics and transcriptomics techniques to further explore the underlying mechanisms.

Metabolomic analysis revealed that H_2_S modulates multiple metabolic pathways in osteoblasts under the HG condition, particularly affecting purine metabolism, glutathione (GSH) homeostasis, glycerophospholipid dynamics, and mineral absorption. Of particular note, we identified the crucial role of H_2_S in regulating GSH metabolism under HG conditions. GSH, a tripeptide composed of glutamine, cysteine, and glycine, serves as a major antioxidant in cells. It plays a critical role in detoxifying ROS. GPX4 is a selenoenzyme that utilizes GSH to reduce lipid hydroperoxides to non-toxic lipid alcohols, thereby preventing lipid peroxidation and ferroptosis (30). The depletion of GSH leads to the inactivation of GPX4, resulting in the accumulation of lipid peroxides and the induction of ferroptosis. In the HG group, significantly elevated pyroglutamic acid and L-5-oxoproline levels indicated GSH metabolic dysfunction, correlating with reduced antioxidant capacity and increased ferroptosis susceptibility. Pyroglutamic acid and L-5-oxproline are intermediates in the GSH cycle; their accumulation indicates disruptions in GSH synthesis and metabolism. Pyroglutamic acid is a byproduct of the γ-glutamyl cycle, which is involved in the synthesis and degradation of GSH. L-5-oxproline is a metabolite formed from the breakdown of GSH and serves as a marker for GSH turnover. Conversely, H_2_S treatment effectively normalized these metabolites, demonstrating its ability to restore glutathione homeostasis. H_2_S has been shown to promote the reduction of glutathione disulfide back to glutathione, thus enhancing the antioxidant capacity of cells (31). These metabolic improvements aligned with restored GPX4 expression and decreased lipid peroxidation, collectively showing H_2_S protects osteoblasts by maintaining redox balance and inhibiting ferroptosis in HG conditions.

Transcriptomic analysis showed that H_2_S upregulated the estrogen signaling pathway and downregulated the P53, PI3K-AKT, and ferroptosis signaling pathways. The latter pathway, ferroptosis, particularly drew our attention. Our study revealed that H_2_S down-regulated the expression of ferroptosis pathway-related genes (*hmox 1* and *ncoa 4*) and up-regulated the expression of *slc40a1*. In the HG condition, *hmox 1* expression was increased, but the presence of H_2_S caused a decrease in its expression. *hmox 1* serves as a crucial enzyme that regulates the speed of heme degradation, which breaks down heme to produce bilirubin, carbon monoxide, and iron (32). Up-regulation of *hmox 1* increases ferritin synthesis, the synthesis of ferritin, leading to changes in the distribution of iron within cells (33). Additionally, *hmox 1* has been identified as a key pathogenic gene related to iron-related ferroptosis in patients with diabetes. The upregulation of *hmox 1* significantly increases iron concentration, ROS production, and lipid peroxidation, consequently facilitating the progression of ferroptosis. Inhibiting *hmox 1* reduces iron concentration and ROS and decreases lipid peroxidation, thereby alleviating ferroptosis caused by diabetes (34). Cells become more vulnerable to ferroptosis due to the transportation of ferritin to autophagosomes for lysosomal breakdown and the liberation of unbound iron facilitated by nuclear receptor coactivator 4 (*ncoa 4*) (35). Recent research suggests that ferroptosis is a form of autophagic cell demise, and the regulation of cellular iron balance through *ncoa 4*-mediated ferritin autophagy enhances the occurrence of ferroptosis (36). Blocking autophagy or knocking out the *ncoa 4* gene can reduce ROS and iron ion concentrations and antagonize ferroptosis. Soluble carrier family 40 member 1 (*slc40a1*), which encodes ferroportin (FPN), is the only iron transporter found in mammals. slc40a1 inhibition reduces intracellular iron output and increases iron concentration, thereby inducing ferroptosis (37). Hao et al. (38) demonstrated that the levels of *slc40a1* expression are decreased in diabetic patients, indicating that *slc40a1* plays a crucial role as a pathogenic gene in the development of iron overload and ferroptosis associated with diabetic cognitive dysfunction.

We demonstrated that HG leads to iron accumulation and increased levels of MDA in osteoblasts. Exogenous H_2_S was found to alleviate iron accumulation and MDA increases caused by HG. GPX4 is a key enzyme in preventing lipid peroxidation and is crucially dependent on glutathione for its activity (39). By maintaining glutathione levels, H_2_S ensures the proper function of GPX4, thereby protecting cells from ferroptosis. Western blot analysis confirmed that H_2_S increased GPX4 and SLC7A11 levels while reducing ACSL4 expression compared to the HG group. SLC7A11 is part of the cystine/glutamate antiporter system, which is critical for maintaining intracellular glutathione levels (40). ACSL4 promotes the formation of lipid peroxides, making its downregulation beneficial in reducing ferroptosis. According to these findings, we further confirmed that ferroptosis caused by HG in osteoblasts results in reduced bone formation, whereas H_2_S can counteract HG-induced osteoblast injury through the ferroptosis pathway.

To examine the impacts of HG on mouse osteoblasts, this research employed novel bioinformatics methods like metabolomics and transcriptomics, alongside *in vitro* cell experiments. The process of HG-induced osteoblast injury is regulated by ferroptosis, an innovative type of programmed cell death. Subsequently, we put forward the novel idea that H_2_S inhibits ferroptosis as a means to combat osteoblast injury caused by HG for the first time. The findings of this study have important implications for fully understanding the pathogenesis of DOP and developing targeted intervention strategies.

While the current findings provide mechanistic insights into H_2_S-mediated protection, this study has several limitations. Most notably, we lack *in vivo* validation in DOP mouse models, which would confirm the physiological relevance of our observed effects on bone microstructure and systemic metabolism. Additionally, while our metabolomic analysis identified altered metabolites, these require targeted spectrometry validation in follow-up studies. Similarly, transcriptomic-identified differentially expressed genes need qPCR and western blot confirmation to verify their roles in the protective mechanism. Future work should also incorporate functional assays like gene knockdown to establish causal relationships beyond correlation.

## Conclusions

**Figure 7:**
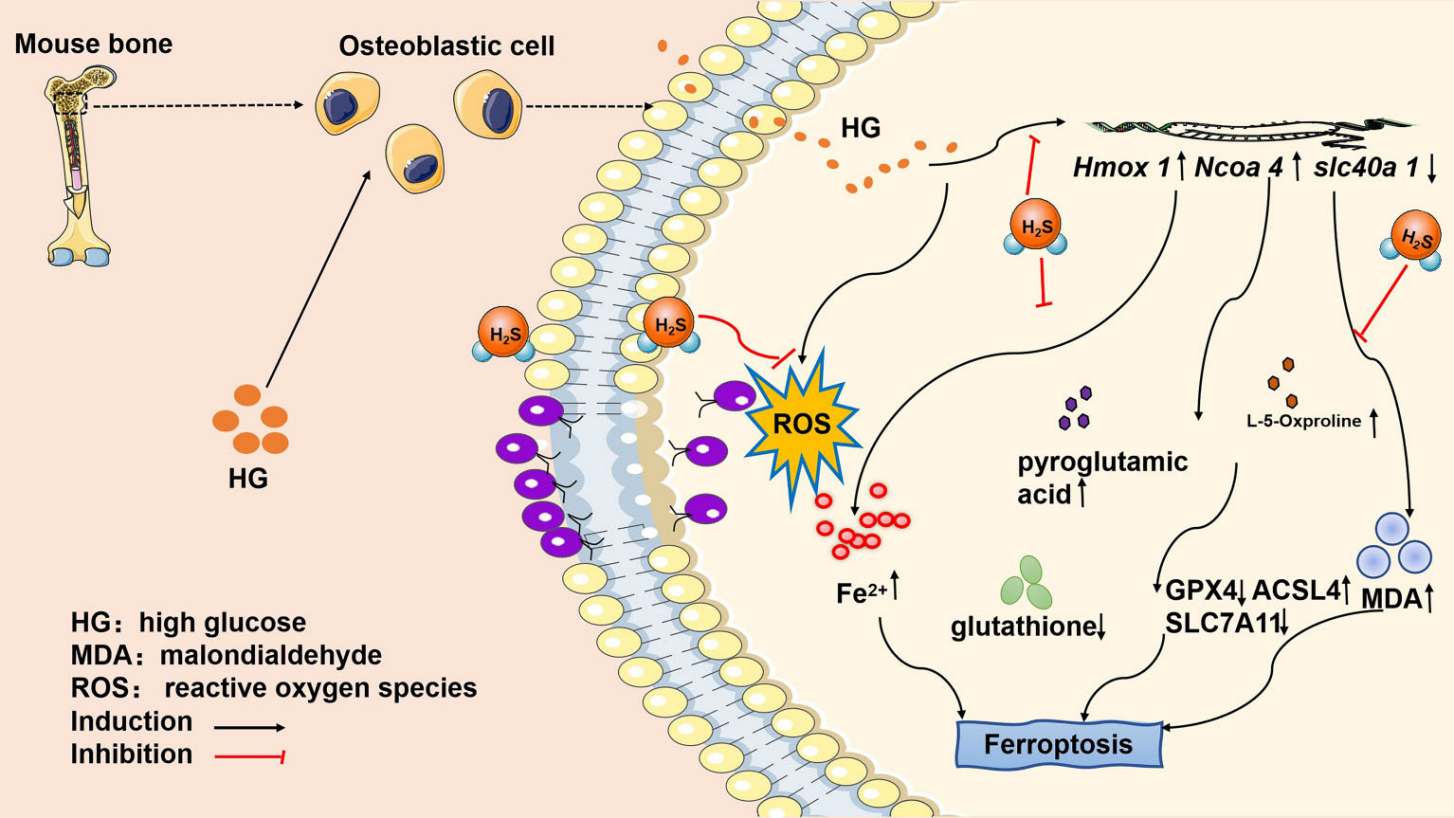
Schematic diagram showing that H_2_S inhibits high glucose (HG)-induced osteoblast injury by inhibiting ferroptosis in diabetic osteoporosis.

This study indicated that ferroptosis is involved in the pathogenesis of DOP and H_2_S effectively alleviated osteoblast injury in DOP by inhibiting ferroptosis (Figure 7). Thus, ferroptosis could be a promising treatment strategy for oxidative stress-related diseases. Additionally, it suggests that H_2_S might serve as a preventive and therapeutic approach to block the progression of DOP.

## Supplementary Materials

Supplementary Material Click to view [pdf].

## Data Availability

All data generated or analyzed during this study are included in this published article.
