# Supplementary material for: H2S inhibits high glucose-induced osteoblast injury by inhibiting ferroptosis in diabetic osteoporosis in vitro
Source: Braz J Med Biol Res. 2025 Nov 14;58:e14679. doi: 10.1590/1414-431X2025e14679 (PMC12645440; doi:10.1590/1414-431X2025e14679)

Figure S1. Analysis of both transcriptome and metabolome in osteoblasts of mice. **A**, The KEGG pathway enrichment analysis. **B**, Network Analysis.

A

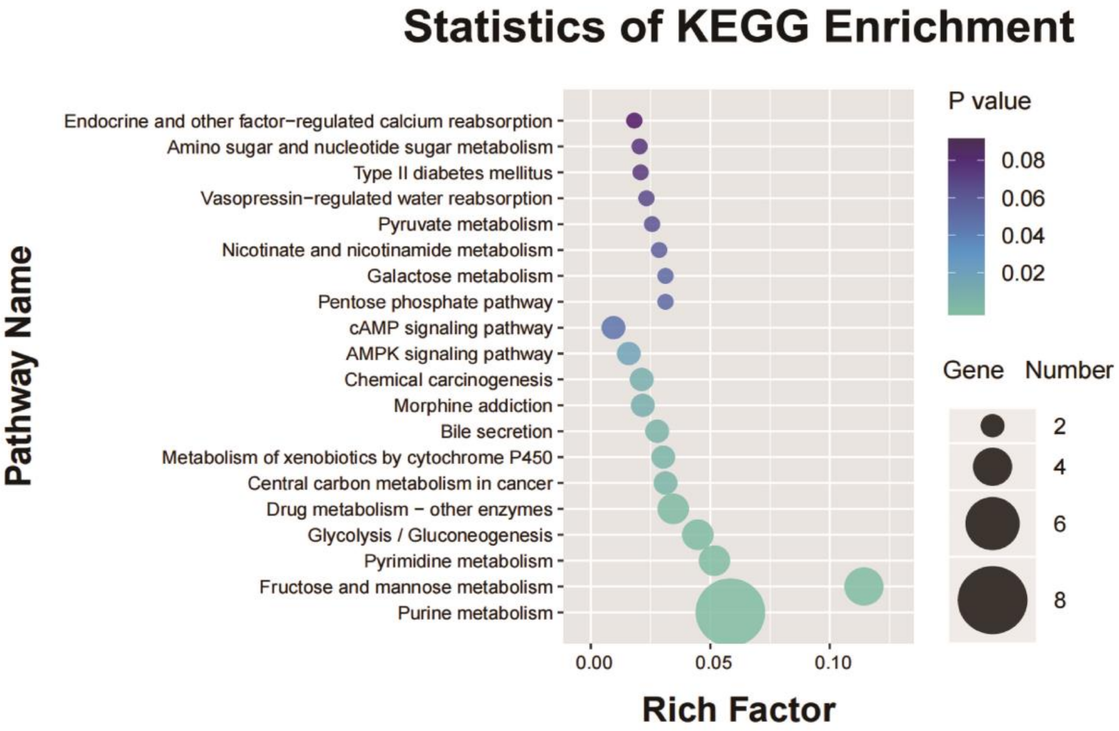

B

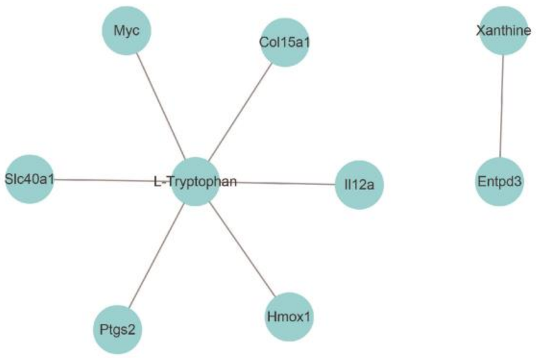

Supplement: Supplementary file 1 [file 1414-431X-bjmbr-58-e14679-suppl.pdf]
